# Ebselen analogues delay disease onset and its course in fALS by on-target SOD-1 engagement

**DOI:** 10.1038/s41598-024-62903-5

**Published:** 2024-05-27

**Authors:** Seiji Watanabe, Kangsa Amporndanai, Raheela Awais, Caroline Latham, Muhammad Awais, Paul M. O’Neill, Koji Yamanaka, S. Samar Hasnain

**Affiliations:** 1 Department of Neuroscience and Pathobiology, Research Institute of Environmental Medicine, Nagoya University, Furo-Cho, Chikusa-Ku, Nagoya, 464-8601 Japan; 2 Molecular Biophysics Group, Department of Biochemistry and System Biology, Institute of System, M0polecular and Integrative Biology, Faculty of Health and Life Sciences, University of Liverpool, Liverpool, L69 7ZB UK; 3 School of Life Sciences, Faculty of Health and Life Sciences, University of Liverpool, Liverpool, L69 7ZB UK; 4 Department of Molecular and Clinical Cancer Medicine, Institute of System, Molecular and Integrative Biology, University of Liverpool, Liverpool, L69 3GE UK; 5 Department of Chemistry, Faculty of Science and Engineering, University of Liverpool, Liverpool, L69 7ZD UK; 6 Institute for Glyco-Core Research (iGCORE), Nagoya University, Nagoya, Japan; 7 Center for One Medicine Innovative Translational Research (COMIT), Nagoya University, Nagoya, Japan; 8 Present Address: Department of Biochemistry, School of Medicine, Vanderbilt University, Nashville, TN 37232 USA

**Keywords:** Amyotrophic lateral sclerosis, Superoxide dismutase, Drug development, Target engagement, Ebselen, Riluzole, Motor neuron disease, Biophysics, Drug discovery, Neuroscience, Structural biology, Medical research

## Abstract

Amyotrophic lateral sclerosis (ALS) selectively affects motor neurons. *SOD1* is the first causative gene to be identified for ALS and accounts for at least 20% of the familial (fALS) and up to 4% of sporadic (sALS) cases globally with some geographical variability. The destabilisation of the SOD1 dimer is a key driving force in fALS and sALS. Protein aggregation resulting from the destabilised SOD1 is arrested by the clinical drug ebselen and its analogues (MR6-8-2 and MR6-26-2) by redeeming the stability of the SOD1 dimer. The in vitro target engagement of these compounds is demonstrated using the bimolecular fluorescence complementation assay with protein–ligand binding directly visualised by co-crystallography in G93A SOD1. MR6-26-2 offers neuroprotection slowing disease onset of SOD1^G93A^ mice by approximately 15 days. It also protected neuromuscular junction from muscle denervation in SOD1^G93A^ mice clearly indicating functional improvement.

## Introduction

Motor neurons are a group of neurons that serve essential function to control voluntary muscle control and contraction. Amyotrophic lateral sclerosis (ALS) is an adult-onset neurodegenerative disease that causes progressive and devastating degeneration of motor neurons in the central nervous system leading to muscle atrophy, paralysis, and fatal respiratory failure with its incidence of 2–3 per 100,000 population per year and a lifetime risk of 1 in 400^1^. Since the identification of *SOD1* as the first causative gene nearly 30 years ago^2^, more than 20 genes have been identified whose mutations result in inherited forms of ALS (familial ALS: fALS)^3–5^, accounting for around 10% of all ALS cases. As clinical surveillance and genomic capabilities have spread, many of the ALS-linked genes have been observed worldwide but with significant geographic variability. For example, an intronic hexanucleotide repeat expansion in chromosome 9 open reading frame 72 (*C9ORF72*) accounts for about 40% of fALS in North America and Europe^6^, while it is rare in the east Asian ALS population^7,8^. Mutations in *SOD1* account for at least 20% of the familial cases and up to 4% sporadic ALS (sALS) in the Western hemisphere while in China they represent around 50% of the fALS cases^5^. Over 180 mutations associated with ALS have been identified for SOD1 including amino acid deletion, insertion, substitution, and premature truncation of the protein. Most SOD1 mutations are inherited in an autosomal dominant manner, with D90A mutation found in both autosomal dominant and autosomal recessive manner. Globally, the most frequent SOD1 gene mutation is D90A^9^ with A4V being the most frequent mutation in the USA^10^ and H46R being the most common mutation in China, Japan and UK^11,12^. In China, very few cases have been reported with A4V or D90A mutations including the single largest recent study in China comprising 923 patients with sALS and 159 with fALS^11^.

SOD1 is a homo-dimeric enzyme with copper and zinc centres responsible for regulating the level of toxic reactive oxygen species particularly in the cytoplasm^13^. Extensive work has established that the pathogenic role of SOD1 in ALS arises from a ‘gain of toxicity’ that leads to the abnormal accumulation and inclusion formation in motor neurons of ALS patients^14–17^. Some point mutations, such as A4V, which are part of the dimer interface, compromise SOD1 dimerization and considerably increase propensity of monomeric protein. Dimer dissociation is a critical aspect for SOD1 destabilisation, leading to the emergence of toxic aggregates in motor neurons^18^. Some mutants result in a ‘gain of interaction’ that was beautifully captured crystallographically for H46R and S134N leading to the linear, amyloid-like filaments and helical, water-filled nanotubes^14^. These arise from exposure of β-sheets leading to non-native conformations that permit dis-allowed interaction between dimers leading to formation of higher order arrays [see Ref.^19^ for a review]. Similar loss of native conformation has been seen in the wild-type SOD1 as a result of a compromise in metal incorporation causing misfolding of wild-type SOD1^16^ and are likely involved in SOD1-associated sALS.

Despite its global presence and a significant lifetime risk, there is no known cure for either sALS or fALS though there are currently more than a dozen clinical trials under different stages (see: https://www.mndassociation.org/research/clinical-trials/treatment-trials). Riluzole and edaravone remain the only two internationally approved drugs for clinical use in ALS treatment with limited benefit. Edaravone, a potent free radical scavenger, approved more recently, has been found to slow disease progression, but its utilisation has remained limited globally due to a variety of factors including its cost and limited benefit. Riluzole modulates the *N*-methyl-*D*-aspartate receptor (NMDA) receptor inhibiting glutamate receptor signalling to reduce glutamate-induced excitotoxicity, but a specific biological target of edaravone has not yet been identified. However, both compounds may also have therapeutic effect due to their antioxidant properties. Recently, the FDA reported a disappointing outcome of a phase 3 trial of a therapy based on transplantation of autologous bone marrow-derived mesenchymal stromal cells, [NurOwn^®^ (MSC-NTF cells)]^20^. Finding therapeutics with good efficacy and safety has remained a challenge in ALS research for both sALS and fALS patients. Given the recent report from China of up to 50% fALS cases arising from SOD1 mutation^5,11^, therapy developed against this well-defined and studied target may prove effective with genetic as well as clinical stratification, and would add to current efforts with Tofersen, an antisense oligonucleotide (ASO) targeting SOD1^21^, whose phase1/2 study results have demonstrated proof-of-concept and proof-of-biology of Tofersen. It has been approved by FDA under the accelerated approval pathway to treat patients. To confirm the clinical benefit of Tofersen, also called Qalsody, a Phase 3 randomized, double-blind, placebo-controlled trial, ATLAS, is ongoing in individuals who are carriers of the SOD1 genetic mutation but do not yet have symptoms^22^.

Many small molecule compounds that interact with SOD1 target have been explored in an attempt to enhance dimer stability and suppress misfolding/aggregation, which ultimately may help to slow disease progression^23^. Ebselen or 2-phenyl-1,2-benzisoselenazol-3(2H)-one, an antioxidant agent, has been studied in clinical trials of hearing loss and bipolar disorder that exhibited good safety profiles in humans^24^. In an early stage ALS drug development campaign, ebselen was shown to restore viability level of mouse neurons expressing human SOD1 mutants and remarkably slowed the disease onset of mice expressing ALS-linked SOD1 mutation^25^. Using structure-based approaches, we have recently discovered next generation ebselen derivatives that improve SOD1 mutant dimer stabilization, enhanced thermal stability and have higher in vitro neuro-protection than edaravone^25–27^. These compounds were designed to penetrate the blood–brain barrier by use of the Pfizer multiparameter optimisation (MPO) approach to CNS drug design^25,28^.

Here, we have taken two of the analogues, MR6-8-2 and MR6-26-2, that are shown to offer the best neuroprotection to ALS SOD1 G93A in transgenic mice experiments and profiled brain and spinal cord specimens. The oral bioavailability and pharmacokinetics were evaluated for one of these compounds for in vivo experiments. The in vitro target engagement of ebselen and its analogues was demonstrated using the bimolecular fluorescence complementation assay confirming the reduction of SOD1 aggregation propensity in human neuroglioma cells by stabilising the dimers of A4V as well as H46R and D90A as evidence for a generic approach for disease-causing destabilised SOD1 dimer. Moreover, the binding of ebselen and its analogues were directly visualised by co-crystallographic experiments with G93A SOD1 providing a direct link to G93A ALS mouse model SOD1^G93A^. The binding poses of these compounds were found to be the same in both A4V and G93A SOD1 structures confirming the generic applicability of these compounds to SOD1 mutants involved in fALS. In vivo experiments of SOD1^G93A^ transgenic mice showed that MR6-8-2 and MR6-26-2 were able to significantly delay the disease onset with improvement of some clinical phenotype indicating significant potential for additional lead optimisation towards candidate selection of a novel therapy for ALS.

## Results

### In vitro neuroprotective activity of ebselen and derivatives in human neuroglioma cells

**Figure 1:**
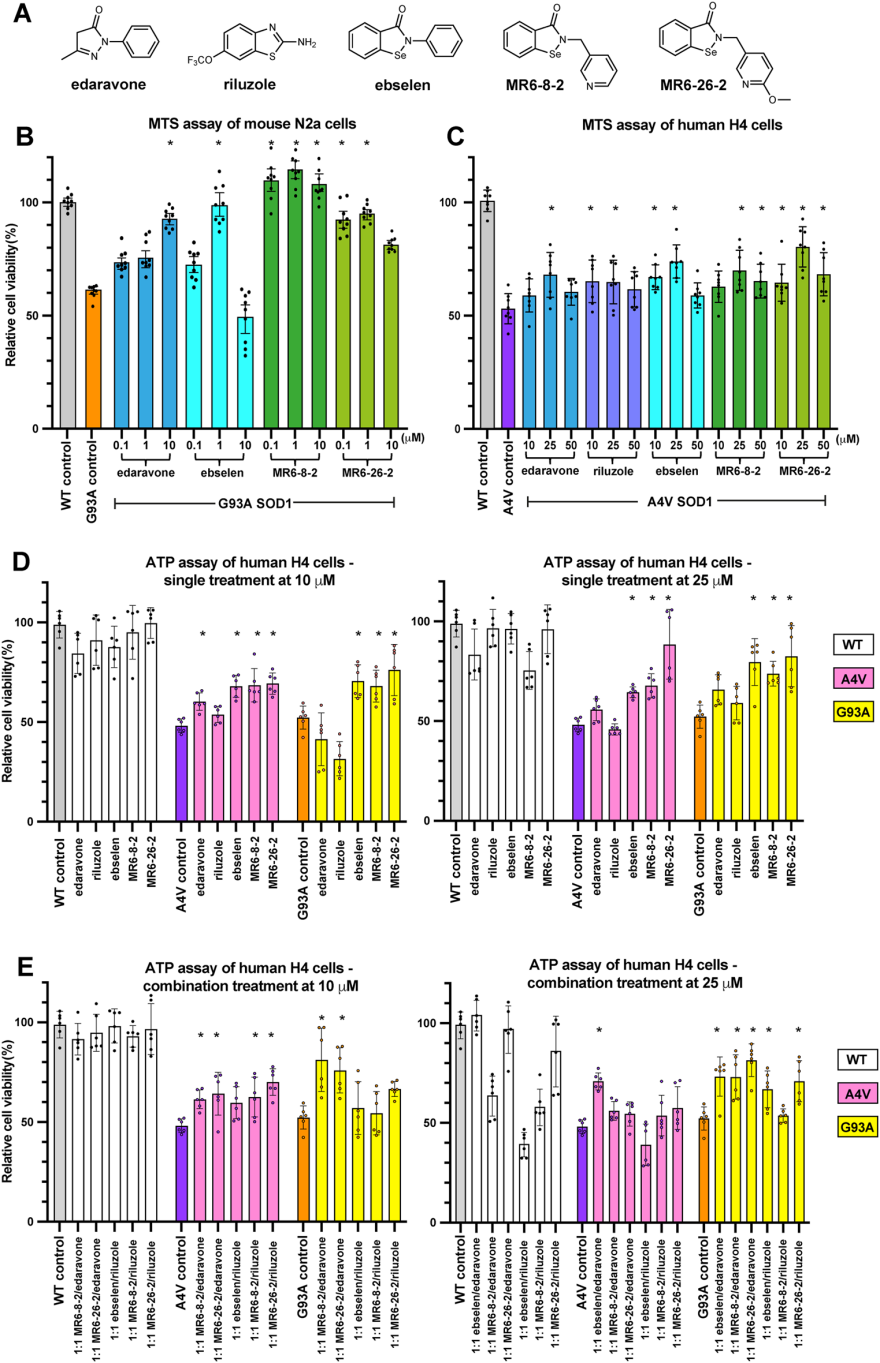
Cell viability of human and mouse neuronal cells expressing wild-type SOD1 and ALS-associated mutants of A4V and G93A under treatments of ebselen-based compounds and ALS-approved drugs. (**A**) Chemical structures of approved ALS drugs, ebselen and derivatives. (**B**) MTS assay of mouse N2a neuroblastoma cells transfected with G93A SOD1. (**C**) human H4 neuroglioma cells transfected with A4V SOD1, and (**D**,**E**) ATP bioluminescent assay for single compound and in combination with approved drugs using human H4 neuroglioma cells transfected with wild-type, A4V and G93A SOD1. Cell viability levels are shown in bar chart with data points and error bars representing standard deviation of the mean from at least 6 separate measurements. Asterisks above bar charts indicate statistically significant improvements (*p* < 0.05) against untreated control groups.

We have previously shown that MR6-8-2 and MR6-26-2 (as Cpd9 and 10, respectively^25^) were able to restore wild-type level viability of mouse neuronal N2a cells expressing G93A SOD1 with a 100-fold lower dosage than edaravone (shown again in Fig. 1B). Mouse neuronal N2a cells were used previously to complement the G93A murine model^25^. To assess if the drugs maintain neuroprotective potency in a human model, we used the human neuroglioma cell line (H4) transfected with wild-type and A4V SOD1 and assessed the cell viability using MTS assay following treatment with ebselen, MR6-8-2 and MR6-26-2 along with two approved ALS drugs—riluzole and edaravone (Fig. 1C). Human cells exhibited the neuroprotective effects at the higher doses of compounds (10–25 μM) compared to the mouse cells (at 0.1–10 μM) (Fig. 1C), while all compounds at 50 μM were found to be toxic with a reduction in the cell viability. All the tested compounds have an antioxidant property with a potential of cross reactivity with the MTS reagent, which may result in false positive outcome, especially at high concentrations of the compounds. To address this concern, we used an alternative ATP bioluminescent assay (CellTiter-Glo) to measure cell viability as a function of ATP in living cells. The transfected cells expressing wild-type, A4V and G93A SOD1 were treated with individual compounds and in combination at two concentrations of 10 and 25 μM for 24 h before determining the cell viability (Fig. 1D,E). About 50% of the cells were found dead in untreated ALS-related mutants (A4V and G93A) compared to wild-type SOD1. The individual compounds at 10 or 25 µM were not toxic to the wild-type SOD1 cells (Fig. 1D). A4V and G93A mutants showed higher cell viability at the dose of 25 μM when treated with individual drugs. Riluzole was least effective followed by edaravone. All organo-selenium drug compounds were significantly better in improving the cell viability than the approved drugs. Edaravone, ebselen, MR6-8-2 and MR6-26-2 increased the cell survival in A4V and G93A mutants at both concentrations. MR6-26-2 was found to be the most effective compound in this series to redeem the viability of cells transfected with A4Vor G93A SOD1 fALS mutation and lower levels of toxicity in wild-type SOD1 cells. To support this in vitro observation, we measured in vitro -dose-response curves of Ebselen, MR6-8-2 and MR6-26-2 at the concentrations between from 2.5 to 100 μM in H4 cells expressing wild-type, A4V and G93A SOD1 (Supplementary Fig. S1). In the wild-type SOD1 cells, all compounds behaved as antagonist with decreasing cellular response over compound doses. Notably, MR6-8-2 gave a sharp fall of viable cells at the concentrations above 25 µM due to significant toxicity to human cells. While in A4V and G93A mutants, all tested compounds showed a bell-shaped dose–response curve with stimulatory effect at the concentrations below 25 µM and toxic effect at higher concentrations. Although MR6-8-2 had greater neuroprotection at concentrations below 25 µM, it turned out to be toxic at lower concentration than other compounds in all of the SOD1 mutants. Ebselen and MR6-26-2 treatment led to high level of cells viability up to 25 µM in A4V and G93A SOD1, before gradually decreasing in cell survival at higher concentrations of the compound. In A4V mutant, MR6-26-2 exhibited the best neuroprotective activity by reviving the cells to maximum level at 12.5 µM with slight drop at 25 µM, while ebselen and MR6-26-2 were identical in their efficiency for G93A SOD1.

To investigate if the combined use of drugs enhances the neuroprotective activity, we also investigated the synergistic effects of ebselen-based compounds and approved ALS drugs using equimolar mixtures at 10 and 25 μM in human H4 cells using the ATP assay (Fig. 1E). In wild-type SOD1, the combined treatments retained the cell viability at the concentration of 10 μM. MR6-8-2 and ebselen induced toxicity in wild-type SOD1 cells at 25 μM. Combination of ebselen/riluzole were also found to be toxic to the wild-type SOD1 cells resulting in cell viability levels even lower than the A4V control group. The combinations of MR6-8-2 with riluzole and edaravone induced toxicity in wild-type SOD1 cells. In A4V mutant, most of the drug combinations increased neuroprotection at 10 μM to a similar level as the monotherapies. The cell viability decreased at 25 μM with the combination of ebselen/riluzole to be least effective. In contrast, neuroprotective activity of combination therapies increased at the higher dose of 25 μM in G93A mutant. Overall, the combined treatment of ebselen-based compounds and ALS drugs did not show synergistic improvement in neuroprotection compared to monotherapy of ebselen-based compounds or ALS drugs.

### In vitro bimolecular fluorescence complementation assay of lead compounds in human neuroglioma cells

**Figure 2:**
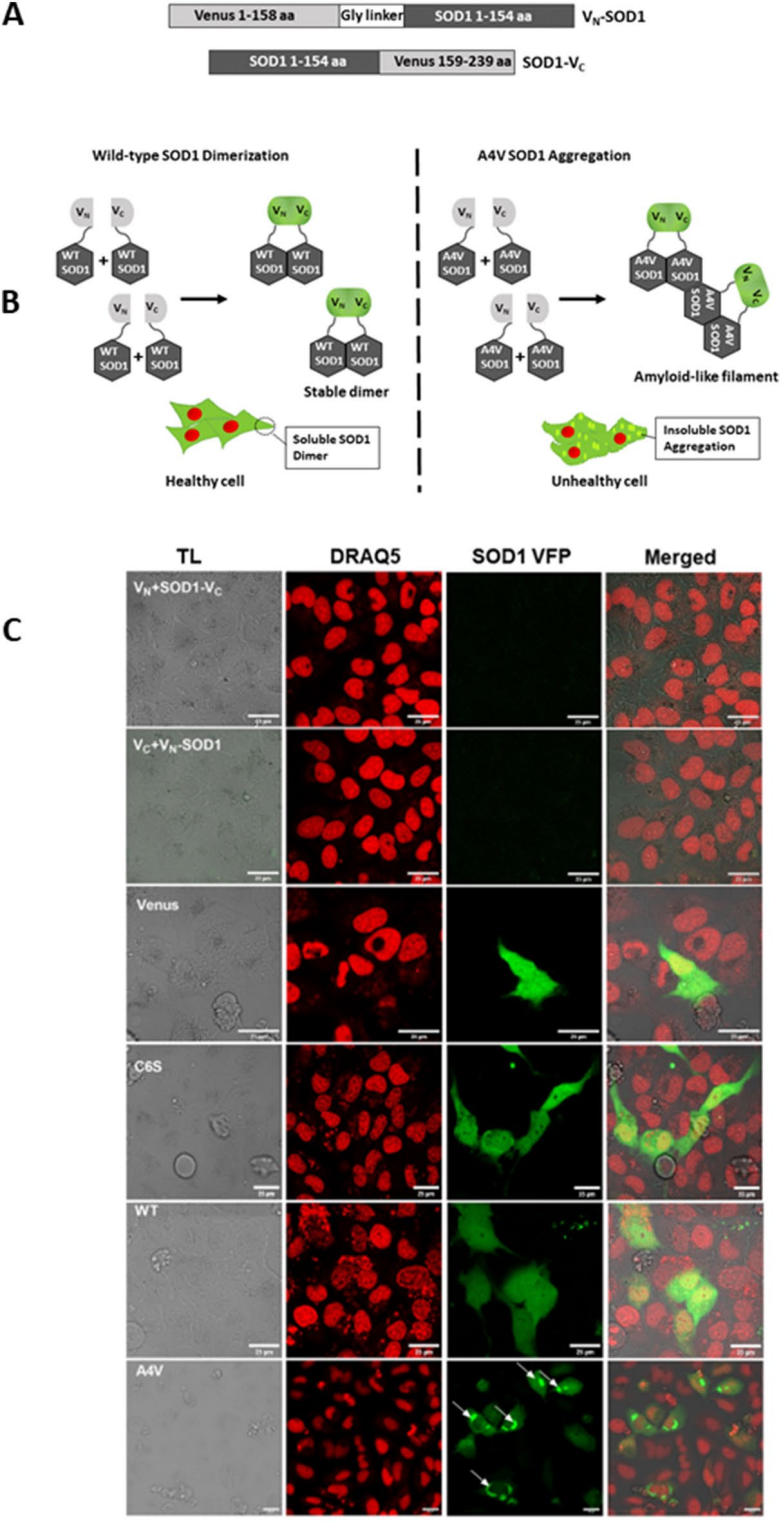
The BiFC assay reliably captures the differences between wild-type and mutant SOD1. (**A**) Schematic illustration of the DNA constructs of the human SOD1 fused to truncated Venus fluorescent protein segments using a larger N-terminal fragment of Venus (V_N_), corresponding to amino acids 1–158, and a smaller C-terminal fragment (V_C_), corresponding to amino acids 159–239. (**B**) Schematic presentation of BiFC assay. Dimerization of WT SOD1 fused to Venus fragments generates fluorescence from the fusion of V_N_ and V_C_ fragments. (**C**) In the A4V SOD1 mutant, Venus fluorescence complementation allows to detect amyloid-like filament formation (aggregates/inclusion) due to non-native (unstable) A4V SOD1 dimerization. Representative images showing the different negative and positive controls to demonstrate the ability of BiFC assay to differentiate between stable, unstable or no SOD1 dimerization. Reciprocal negative controls of SOD1 V_C_ with empty V_N_ (V_N_ + SOD1 V_C_ or V_N_ SOD1 with empty V_C_ (V_C_ + V_N_ SOD1) vectors showing no fluorescence due to lack of dimerization in the absence of complimentary SOD1. Venus is pVenus N1 empty plasmid that does not contain SOD1 and only expresses VFP; C6S, a positive control, is an internal non-specific mutation and interaction of V_N_ C6S SOD1 and C6S SOD1 V_C_ presents uniform fluorescence WT represents the interaction of V_N_ WT SOD1 and WT SOD1 V_C_ displayingeven fluorescence A4V, aSOD1 mutant and represents the interaction of V_N_ A4V SOD1 and A4V SOD1 V_C_ leading to the aggregates/inclusion formation (shown by white arrows), scale bar: 25 μm. Left to right panel for each control shows transmitted light (TL), DRAQ5 stained nuclei, SOD1 VFP (after complementation of SOD1 Vc or SOD1 V_N_) and merged images respectively.

Aggregation of the SOD1 mutant represents common factors of SOD1-linked fALS. The cellular assay that monitors the reduction of an aggregating ability of mutant SOD1 following the treatment with drugs can offer a powerful tool to assess on-target engagement. Here, we used BiFC assay^29^, based upon the interaction between at least two proteins to produce fluorescence, each protein fused to non-fluorescent, truncated complementary fragments of a fluorescent reporter^30^, which in this case is VFP (Fig. 2A,B). Using this phenomenon together with confocal fluorescence microscopy, the BiFC assay could help identify the presence of regular dimers as well as inclusion bodies or aggregates produced from destabilised SOD1 mutant dimer or from monomerization (Fig. 2C). The BiFC assay has been shown previously to capture the aggregation propensity of mutant SOD1^29^. Here we aim not only to detect the inclusion bodies but also to evaluate the reduction of aggregation propensity of SOD1 resulting from the reversal of pathological SOD1 to the wild-type phenotype following engagement of SOD1 by the drugs. The functional VFP once formed from V_N_ and V_C_ is stable and doesn’t dissociate easily and may result in the self-assembly^31^ leading to the false positive results. To rule this out, various negative and positive controls were used (Fig. 2C). The cells transfected with either V_N_-SOD1 and empty V_C_ vectors or with empty V_N_ and SOD1-V_C_ did not display any fluorescence due to the absence of SOD1 dimeric partner that is needed for complementation of Venus (V_N_–V_C_) fragments (Fig. 2C V_N_ + SOD1-V_C_ and V_C_ + V_N_-SOD1). The full-length Venus plasmid, without SOD1, showed diffused fluorescence evenly distributed in the cytoplasm as well as in the nucleus (Fig. 2C-Venus) due to absence of SOD1localization signal. H4 neuroglioma cells transfected with WT or C6S (background mutation generated previously to have only Cys111 for binding for DSF studies^23^) SOD1 showed uniform fluorescence in the cytosol without any aggregation due to the stable dimerization of wild-type or C6S SOD1 (Fig. 2C C6S, WT) . In contrast, cells expressing mutant A4V SOD1 displayed fluorescent aggregates that represent SOD1 inclusions (Fig. 2C, A4V-white arrows).

Following the validation of the BiFC assay through the use of various controls, the H4 cells were transfected with different mutant SOD1 variants (A4V, G93A, H46R and D90A) and live cell imaging was carried out under non-treated and drug-treated conditions. These mutations were chosen to cover a range of features including disease onset, duration and location of mutational site in the SOD1 structure as well as to complement the well-established murine model, G93A. A4V represents a mutation at the dimer interface, known to cause dimer destabilisation, is the most frequently occurring ALS SOD1 mutation in North America and causes shortest disease course from the onset of disease. H46R located on β-strand 4 represents a mutation in the catalytic pocket but has a very long disease course which can last more than 20 years despite the loss of enzymatic activity. D90A is globally the most frequent SOD1 gene mutation and is the only known ALS patients homozygous for the D90A mutation found primarily in Sweden and Finland^32^.

**Figure 3:**
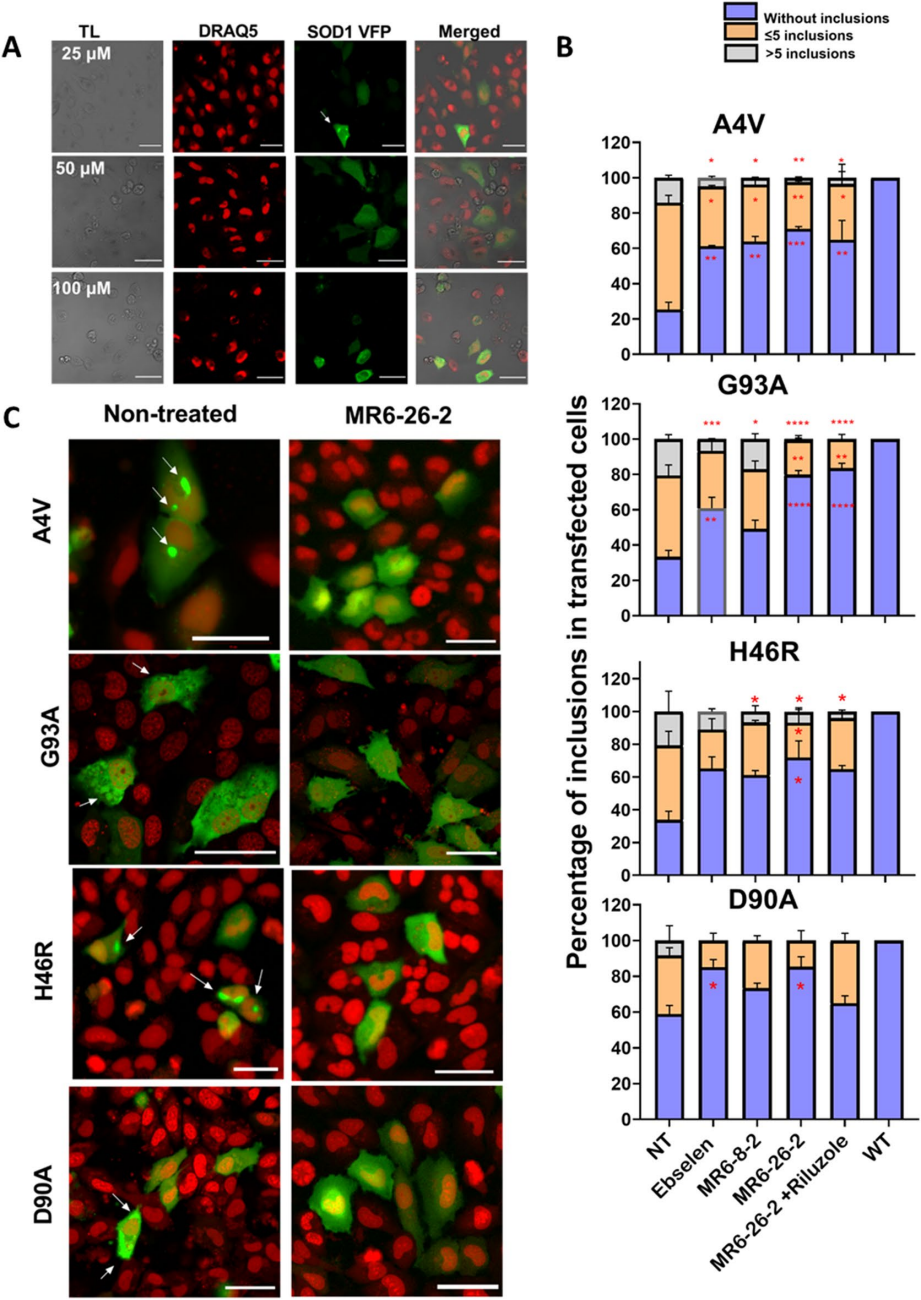
Ebselen and its derivatives reduce aggregating species in human cell model. (**A**) Effects of drug (MR6-26-2) doses 25, 50 and 100 µM on SOD1 dimerization following transfection and drug treatment of H4 cells with A4V (V_N_ + V_C_), scale bar: 25 µm. Left to right panel for each dose shows transmitted light (TL), DRAQ5 stained nuclei, SOD1 VFP (after complementation of SOD1 V_C_ or SOD1 V_N_) and merged images respectively. (**B**) Quantification of the number of inclusions per cell. 50–200 cells were counted per condition and per experiment and classified into three groups: blue, orange, and grey bars represent the percentage of cells without inclusions, with 5 or less inclusions and cells with more than 5 inclusions respectively. Data are expressed as mean ± SD of at least three independent experiments. One-way ANOVA, with Dunnet’s multiple comparison, was used for statistical analysis with significance level of * p < 0.05 ***p < 0.001 or ****p < 0.0001, which represents statistically different results between mutant SOD1 with and without compound treatment. (**C**) Representative images showing reduction in the aggregation propensity of H4 cells expressingA4V, G93A, H46R and D90A following treatment with most effective MR6-26-2 compound versus non-treated cells (scale bar: 50 µm). White arrows indicate the mutant SOD1 aggregation. The scaling of the images has been kept different to make the aggregates/inclusion bodies clearly visible in non-treated conditions, and to show the maximum possible number of cells within a single field as proof of drug efficacy in stable SOD1 dimerization and uniform fluorescence following treatment with MR6-26-2.

Although the viability assay showed 10 or 25 µM to be an optimum drug dose for MR6-26-2, 50 µM drug concentration was found to reduce the aggregates effectively (Fig. 3A). A further increase in drug concentration to 100 µM resulted in cytotoxicity (Fig. 3A). Therefore, 50 µM drug concentration was used to investigate the effect of drugs on aggregation propensity in BiFC assay. Following the image analysis, the automated quantification of inclusion bodies was carried out for A4V, H46R and D90A. G93A SOD1 expressing cells displayed fibrous aggregates unlike defined rounded and bright inclusion bodies displayed by other SOD1 variants (Fig. 3C). G93A aggregates were therefore counted manually as cell profiler was unable to detect the aggregates of varying sizes accurately.

The image analysis and quantification of aggregates/inclusions showed the variable number of inclusions per cell and were therefore grouped into three categories—no inclusion; ≤ 5 and > 5 inclusions (Fig. 3B)^33^. On comparing the fractions of inclusions between different SOD1 variants, A4VSOD1cells displayed significantly high percentage of ≤ 5 as well as > 5 inclusions per cell compared to cells with no inclusions (Fig. 3B, NT, 2-way ANOVA, p < 0.001). In G93A and H46R, the cells with ≤ 5 were significantly higher than > 5 inclusions (2-way ANOVA, p < 0.01). In D90A variant however, cells with no inclusions were predominant and were significantly higher compared to cells with ≤ 5 or > 5 inclusions (2-way ANOVA, p < 0.001). When treated with ebselen and its second-generation analogues MR6-8-2 and MR6-26-2, all the compounds were able to engage the SOD1 target. All drugs significantly reduced the propensity of aggregation in A4V and G93A in particular as demonstrated by the reduction in the number of cells displaying inclusions as well as the number of inclusions within the cell (Fig. 3A,B) [1-way ANOVA, p < 0.001]. MR6-26-2 was found to be the most effective in reducing the aggregates in all variants of mutant SOD1 (A4V, G93A, H46R and D90A) with significantly higher percentage of cells displaying no inclusions compared to that of the untreated mutant SOD1 cells [one-way ANOVA, p < 0.05–0.0001] (Fig. 3B). The mutant SOD1 Venus cells treated with ebselen and MR6-8-2 also showed significant reduction in aggregating propensity of cells but was not as effective as MR6-26-2. The effects of ebselen and MR6-8-2 also varied in different mutant SOD1 variants with being least effective in H46R and D90A (Fig. 3B). In addition to a reduction in number of cells displaying aggregates, the drug treatment also caused a significant reduction in the number of inclusions within the cells. The effect was most prominent with MR6-26-2 treatment which significantly reduced the number of inclusions per cell (both < 5 and > 5). With MR6-26-2 treatment, no cells were detected with more than 5 inclusions per cell for G93A and D90A SOD1 variants and for A4V SOD1 only 29% cells were detected that displayed more than 5 inclusions per cell compared to 75% in A4V untreated cells (Fig. 3B). Using the combination of MR6-26-2 with riluzole similar effect to MR6-26-2 was observed and the combined use did not induce any synergistic effect on the reduction of SOD1 aggregation propensity (Fig. 3B). The mutant SOD1 cells treated with most effective drug i.e. MR6-26-2 (Fig. 3C) appeared healthy with uniform fluorescence similar to WT cells (Fig. 2C).

### Crystallographic studies of G93A SOD1 with ebselen and derivatives

**Figure 4:**
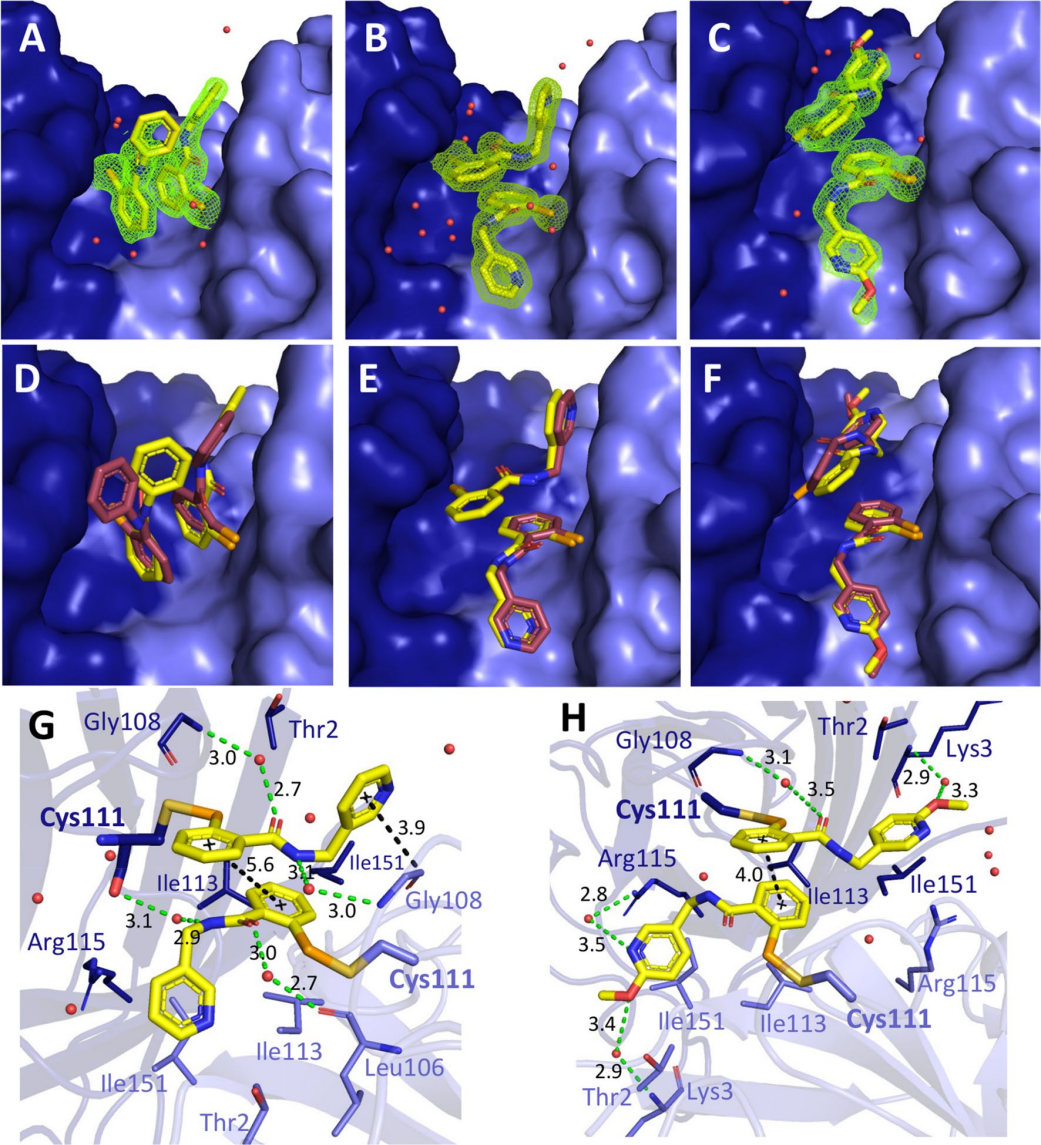
Co-crystalised structures of G93A SOD1 with ebselen, MR6-8-2 and MR6-26-2. Electron density (2F_o_–F_c_) maps of (**A**) ebselen, (**B**) MR6-8-2, and (**C**) MR6-26-2 are contoured at 1σ in green mesh. Ligands and waters are illustrated as yellow sticks and red sphere, respectively. Individual SOD1 monomers are coloured in dark and light blue surface. Overlaid ligand poses in co-crystalised G93A (yellow sticks) and A4V SOD1 (pink sticks) structures of (**D**) ebselen, (**E**) MR6-8-2, and (**F**) MR6-26-2. Structures of A4V SOD1 with corresponding compounds are obtained from PDBs: 6Z4G, 6Z4L and 6Z4M respectively. Binding modes of (**G** MR6-8-2 and (**H**) MR6-26-2 are shown among amino acid residues (dark and light blue sticks) and water molecules in SOD1 dimer interface. Hydrogen bonds and distances between molecules are shown as green and black dashes, respectively. Numbers represent distances in Ångstroms.

MR6-8-2 and MR6-26-2 are the compounds that were developed from the previous generation of ebselen scaffold based compounds by optimising the interactions observed in the co-crystalised A4V SOD1 structures with the first generation compounds reported previously^25^. Both compounds have excellent activity in stabilising the A4V SOD1 dimer and exhibit greater neuroprotection in transfected mouse and human neuron cells than approved ALS drugs. It is important to validate therapeutic activities of MR6-8-2 and MR6-26-2 for in vivo disease progression in the experimental mouse model of ALS. Mice expressing human A4V SOD1 develop ALS symptoms in very late stage unlike in humans where A4V SOD1 shows aggressive ALS with early onset, and as such are not considered a good model for human A4V fALS disease^34^. Thus, G93A SOD1 transgenic mice have despite their limitations have remained the ‘gold standard’ model for human disease and are more commonly used as the in vivo model to evaluate disease development in ALS studies. To ensure that ebselen-based compounds do not have different binding behaviour in the G93A mutant compared to A4V, we solved the high-resolution structures of G93A SOD1 co-crystalised with ebselen, MR6-8-2 and MR6-26-2 (Fig. 4). The statistics of data collection and structure refinement are given in Supplementary Table S1 together with their PDB codes. There was clear electron density of ligands at cys111 of dimer interface of G93A SOD1 allowing unambiguous modelling of MR6-8-2 and MR6-26-2 (Fig. 4A–C). The overlaid high-resolution P2_1_ structures of G93A and A4V SOD1 co-crystalised with corresponding compounds demonstrated that no significant change in binding modes of the compounds between both mutants (Fig. 4D–F). Similar to co-crystalised A4V SOD1, the pyridyl tail of MR6-8-2 and MR6-26-2 was placed in opposite directions and packed into the gap between N- and C-termini of G93A SOD1 (Fig. 4G,H). One of MR6-8-2 molecule in SOD1 dimer was seen to form the bridge with Gly108 of another monomer giving better stabilisation than ebselen while this style of binding was not observed in MR6-26-2. The methoxy group present in MR6-26-2 would make the molecule too large for bridging interactions and it prefers to fit into the gap between Thr2 and Ile151. Moreover, modification of cys111 by the ebselen-based compounds does not influence the overall conformation of G93A SOD1 compared to the ligand-free structure with RMSD values of 0.16–0.25 (Supplementary Fig. S2A). Only the loop (residues 106–112) at dimer interface opens wide on compound binding, especially MR6-8-2 shifts it furthest due to cross-monomer binding pose (Supplementary Fig. S2B). This crystallographic evidence confirms that ebselen-based compounds have similar molecular functions in both A4V and G93A SOD1 where they help to stabilise the dimer. These structures confirm on-target engagement of these compounds and should thus be translatable to in vivo SOD1^G93A^ mice studies. In contrast, neither riluzole nor edaravone, the only approved drugs in clinical use globally, are found to be present in the structures obtained through co-crystallisation with these drugs. This is to be expected as neither of these drugs target SOD1.

### MR6-8-2 and MR6-26-2 delayed disease onset and decreased misfolded SOD1 in SOD1^G93A^ mice

**Figure 5:**
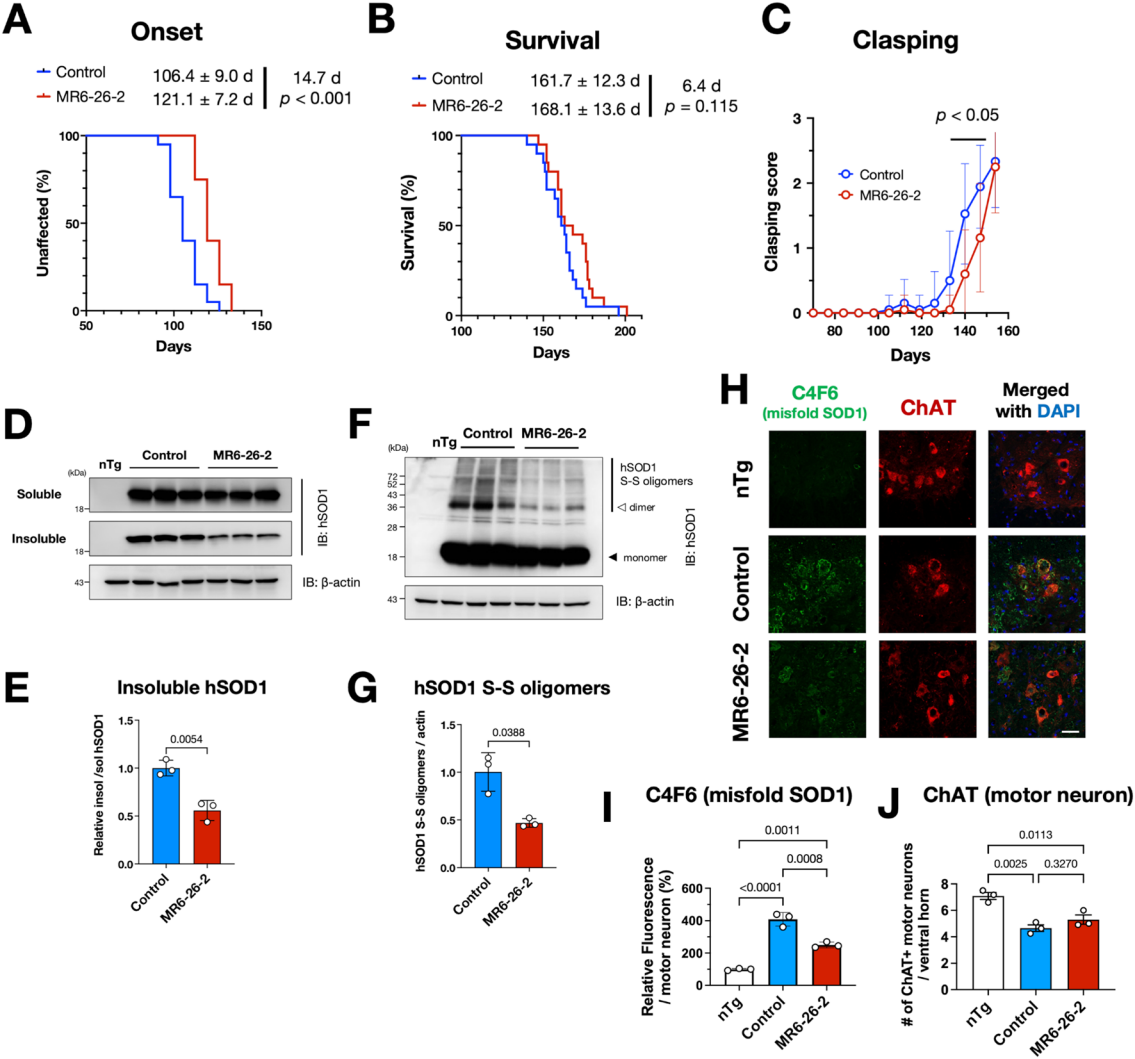
MR6-26-2 decreased misfolded SOD1 species and delayed the disease onset of SOD1^G93A^ mice. (**A**,**B**) Onset (**A**) and survival (**B**) curves of the control or MR6-26-2 treated female SOD1^G93A^ mice plotted over time (n = 20, each). The mean ages for onset or survival are shown with SD. (**C**) Changes in clasping score of the control or MR6-26-2 treated SOD1^G93A^ mice plotted over time (n = 20, each). The data were analyzed by two-way ANOVA following posthoc multiple comparisons with Šidák correction. (**D**,**E**) Decrease of insoluble SOD1 species in the spinal cords of MR6-26-2 treated SOD1^G93A^ mice at 120 days old. A representative immunoblotting image is shown (**D)**, and the relative expressions of insoluble SOD1 per soluble SOD1 were quantified and plotted as mean ± SEM (**E**). (**F**,**G**) SOD1 oligomers produced by aberrant disulfide bonds (hSOD1 S-S oligomers) were decreased in the spinal cords of MR6-26-2 treated SOD1^G93A^ mice at 120 days old. A representative immunoblotting image indicates the hSOD1 S-S oligomers (a bold line) (**F**). Relative expressions of hSOD1 S-S oligomers were quantified and plotted as mean ± SEM (**G**). (**H**–**J**) Immunofluorescent analyses of lumbar spinal cord sections of early symptomatic SOD1^G93A^ mice showed the decrease of misfolded SOD1 species, detected using a C4F6 antibody that specifically recognizes misfolded SOD1, by the MR6-26-2 treatment. Motor neurons were identified using an anti-choline acetyltransferase (ChAT) antibody. Representative immunofluorescent image is shown (**H**), and the relative fluorescence intensity of C4F6 **(I)** or the number of ChAT-positive motor neurons **(J)** in the ventral horn averaged per mouse was quantified and plotted as mean ± SD. Each dot represents the section used for the quantification (**I**,**J**). Scale bar: 50 µm.

To evaluate neuroprotective potential of these second generation ebselen-derivative compounds in vivo, we performed survival experiments of SOD1^G93A^ transgenic mice with chronic oral administration of MR6-8-2 or MR6-26-2. The mice were fed by powder food mixed with/without 0.016% w/w MR6-8-2 or MR6-26-2 (estimated dose: 24 mg/kg) from 70 days of age to the disease end stage. We found the significant delay in disease onset of SOD1^G93A^ mice administrated with MR6-8-2 or MR6-26-2 (control: 104.4 ± 8.7 days vs MR6-8-2: 115.5 ± 9.2 days; control: 106.4 ± 9.0 days vs MR6-26-2: 121.1 ± 7.2 days, shown as mean ± SD) (Fig. 5A, Supplementary Fig. S3). Although survival time of MR6-26-2 treated SOD1^G93A^ mice did not show a significant extension, improvement of clasping scores was observed (Fig. 5B,C), though the body weights and rotarod performance were unaffected (Supplementary Fig. S4). The in vivo role of ebselen-derivative in stabilising SOD1 dimer and decreasing misfolded SOD1 proteins was evaluated in the spinal cords of compound-treated SOD1^G93A^ mice at the early symptomatic stage. Insoluble SOD1 proteins and SOD1 oligomers with aberrant disulfide bonds were sharply decreased in the spinal cords of MR6-26-2 treated SOD1^G93A^ mice (Fig. 5D–G). MR6-26-2 treatment also reduced the accumulation of misfolded SOD1, detected by the antibody specific to misfolded SOD1 (C4F6, Fig. 5H,I) without affecting the motor neuron degeneration (Fig. 5J).

### MR6-26-2 improved proteostasis and a neuromuscular junction pathology of SOD1^G93A^ mice

**Figure 6:**
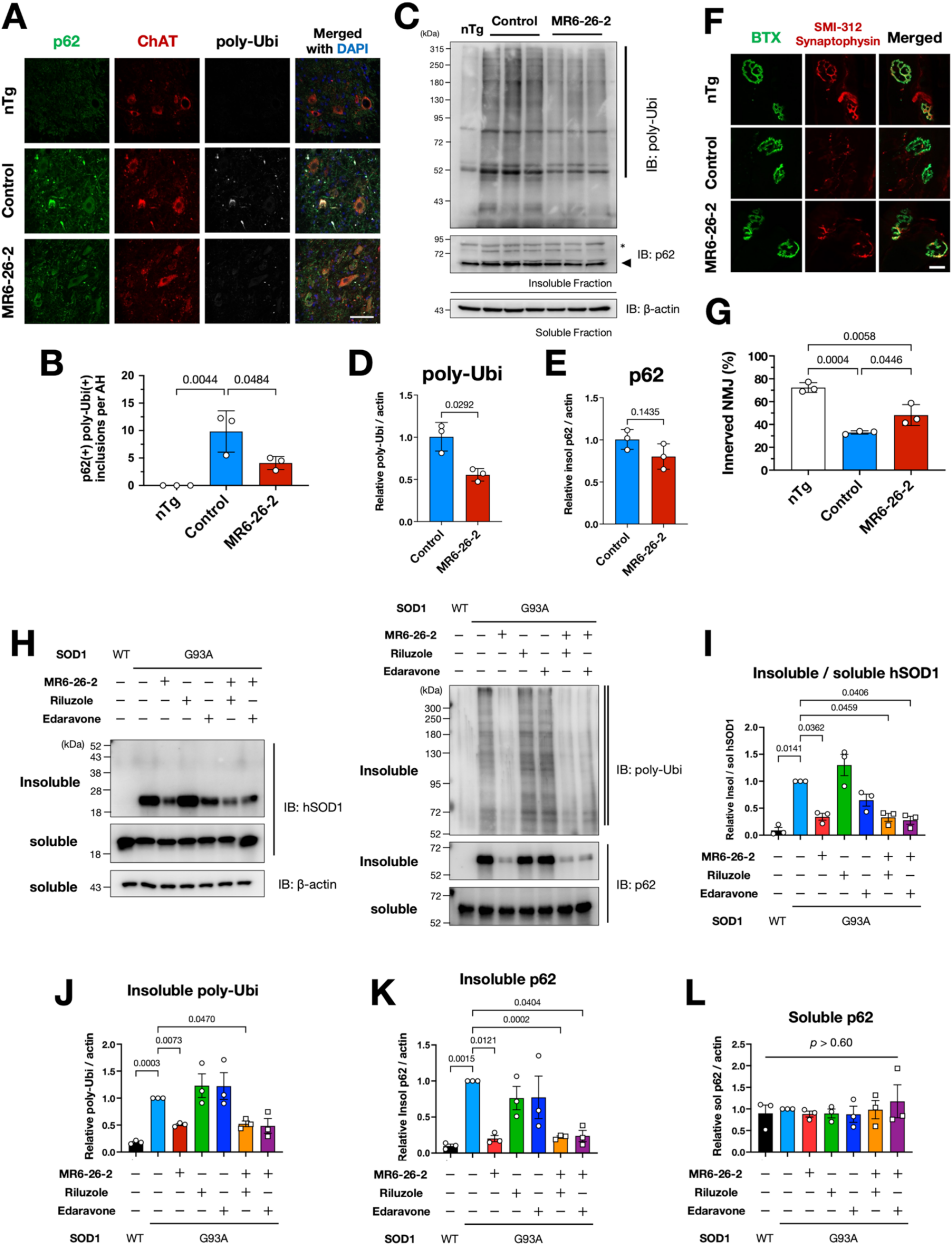
MR6-26-2 ameliorated a neuromuscular junction pathology of SOD1^G93A^ mice and improved proteostasis in vitro and in vivo*.* (**A**,**B**) The MR6-26-2 treatment reduced poly-ubiquitin- (poly-Ubi) and p62/SQSTM1-positive inclusions in the 120-days-old SOD1^G93A^ lumbar spinal cord. Representative immunofluorescent images are shown (**A**). The number of the inclusions per anterior horn (AH) quantified from three mice are plotted as mean ± SEM (n = 3) (**B**). Scale bar: 75 µm. (**C**–**E**) Immunoblot analyses of poly-Ubi and p62 expressions in insoluble fractions of spinal cords from the control or MR6-26 treated SOD1^G93A^ mice at 120 days old. Representative immunoblotting images are shown (**C**), and the quantification results of insoluble poly-Ubi (bold line in **C**) and insoluble p62 (black rectangle) are expressed as mean ± SEM in (**D**) and (**E**), respectively. An asterisk indicates non-specific bands. (**F**,**G**) MR6-26-2 reduced denervation at neuromuscular junctions (NMJs) of 120-days-old SOD1^G93A^ mice. Representative immunofluorescent images of tibialis anterior muscles are shown (**F**). Bungarotoxin (BTX) and synaptophysin indicate postsynaptic acetylcholine receptors on muscles and motor nerve terminal ends, respectively. Innerved NMJ ratio was quantified as a co-localization ratio of BTX and synaptophysin and plotted as mean ± SD (n = 3) (**G**). (**H**–**L**) MR6-26-2 improved proteostasis in cultured Neuro2a cells. Representative immunoblotting images are shown (**H**), and the quantification results of insoluble SOD1 ratio against soluble SOD1 (**I**), insoluble poly-Ubi (indicated by a double line in **H**) (**J**), and insoluble (**K**) or soluble (**L**) p62 are expressed as mean ± SEM (n = 3, each), respectively. Three independent experiments were performed, and the amount of each protein was quantified as relative to the DMSO-treated controls with SOD1^G93A^ expression. Scale bar: 10 µm.

Proteostasis defects are deeply associated with pathological mechanism of neurodegenerative diseases. Therefore, we assessed proteostasis in the MR6-26-2 treated SOD1^G93A^ mice. Abnormal accumulation of poly-ubiquitinated proteins and aggregates of p62, a poly-ubiquitin binding protein, are indicative of defective proteostasis. Immunofluorescence analysis revealed that p62- and poly-ubiquitin-doubly positive inclusions were mainly observed in the ChAT-positive motor neurons and that those inclusions were also substantially reduced by the MR6-26-2 administration (Fig. 6A,B). Immunoblotting also confirmed that the level of poly-ubiquitinated proteins was decreased in the spinal cords of SOD1^G93A^ mice (Fig. 6C,D). Insoluble p62 proteins were not affected by the compounds (Fig. 6E). These data suggest that MR6-26-2 partially improves proteostasis in the spinal cord of SOD1^G93A^ mice by facilitating the proper folding of mutant SOD1. We also analysed a neuromuscular junction pathology, since ebselen-derivative showed functional improvement of SOD1^G93A^ mice and proteostatic modulation rescued glutamatergic synaptic dysfunction in *Drosophila* neuromuscular junction^35^. Quantitative analysis of neuromuscular junctions in tibialis anterior muscles revealed that MR6-26-2 administration significantly ameliorated the denervation of skeletal muscles in SOD1^G93A^ mice (Fig. 6F,G). Moreover, similar improvement in proteostasis by MR6-26-2 was confirmed in cultured neuronal cells (Fig. 6H–L). MR6-26-2 treatment reduced poly-ubiquitinated proteins (Fig. 6J) or insoluble p62 (Fig. 6K) as well as insoluble SOD1 species (Fig. 6I). Riluzole, one of the approved drugs for ALS, showed no effect on proteostasis. Edaravone, another approved agent for ALS, showed a tendency to reduce insoluble SOD1 but had little effect on poly-ubiquitinated proteins or insoluble p62. Overall, these experiments have demonstrated an efficient improvement in proteostasis through stabilisation of SOD1 mutants’ dimeric assembly and amelioration of disease by the oral administration of ebselen-derivative compounds in SOD1^G93A^ mice.

### Bioavailability and pharmacokinetic evaluation of MR6-26-2

**Figure 7:**
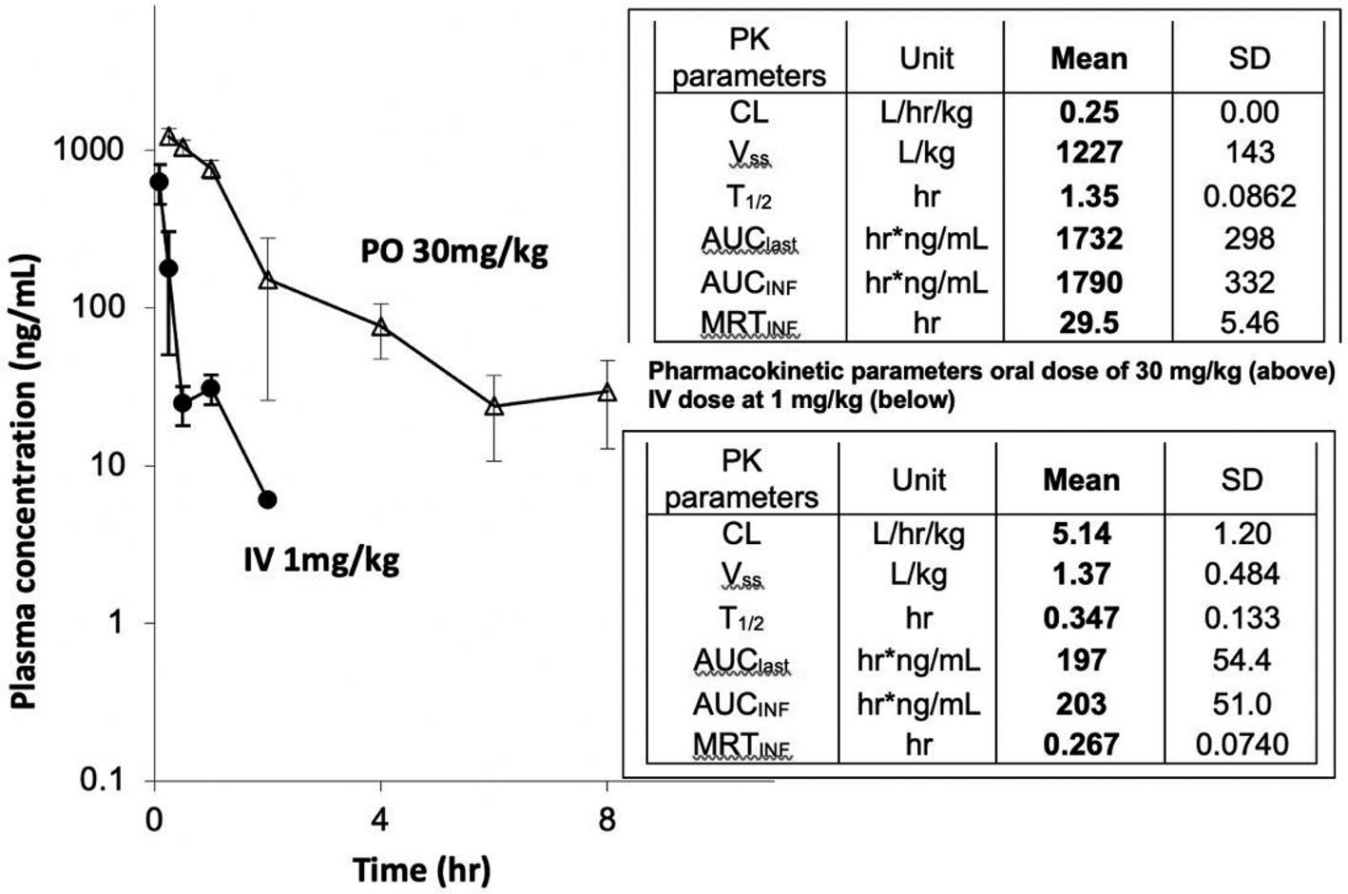
Mean plasma concentration–time profiles and Pharmacokinetic parameters of MR6-26-2. Mean plasma concentration–time profiles of MR6-26-2 after single IV and PO dose administrations in female CD1 mice. Pharmacokinetic parameters of MR6-26-2 after an oral dose of 30 mg/kg (top table) and an IV dose at 1 mg/kg (lower table) in female CD1 mice. F = (AUC_INF_-PO/mean AUC_INF_-IV)/(Dose_PO_/Dose_IV_) × 100%, AUC_last_ was alternatively used for F calculation when AUC_INF_ was not available or beyond 120% of AUC_last_. The compound were formulated in 5%DMAC + 95% (20% HP-beta-CD in saline) at 0.2 mg/mL for IV dosing and CMC (0.5%), benzyl alcohol (0.5%), Tween 80 (0.4%) and NaCl (0.9%) at 3 mg/mL for PO dosing, respectively. PK parameters were estimated by non-compartmental model using WinNonlin 8.2.

We tested the oral bioavailability of one of the more potent compounds, MR6-26-2, in mice and results of a pharmacokinetic (PK) study are provided in Fig. 7 showing the plasma exposures following intravenous (IV) or oral administration. At a single oral dose of 30 mg/kg in female CD1 mice, MR6-26-2 was shown to have 29% oral bioavailability with a short plasma half-life of 1.5 h with fast clearance. The analysis is based on free measurable amounts of free drug in the plasma and thus is likely to be an underestimation of active drug compound due to the extensive serum albumin binding seen with drugs in this class. The actual concentration of drug available to engage the target is likely to be much higher than revealed in this PK study with previous work showing that ebselen can be released from its cysteine binding site on albumin by interaction with glutathione of other protein thiols resulting in complex exchange and release behaviour^36^.

## Discussion

ALS is a devastating neurodegenerative disease with no effective treatment including the clinically approved drugs riluzole and edaravone^37^. Limited options and efficiency of available ALS medications make development of new therapies essential that are aimed at slowing down the disease progression, improving the quality of life and extending life of an ALS patient. Combination therapies using two or more drugs have been considered as possible strategies to develop ALS remedies^38–40^. Tofersen is the only FDA approved drug under the accelerated approval pathway specifically for treating SOD1-related ALS patients. It reduces the accumulation of SOD1 mutant aggregates by inhibiting mRNA of SOD1 mutant thus restricting the production of the protein^22^. Prevention of cytosolic destabilisation and aggregation of SOD1 in motor neurons is a clear strategy towards finding a suitable treatment for SOD1 fALS patients. Extensive work has established that destabilisation of dimer and formation of aggregates lead to accumulation of toxic protein inclusions in neurons. We have developed potent ebselen-based compounds, MR6-8-2 and MR6-26-2 that stabilise dimers of ALS-associated mutants of SOD1 and have validated the neuroprotective activity in a mouse cell-based model expressing G93A SOD1 mutation^25^.

In this work, we assessed in vitro neuroprotective activity of these ebselen-based compounds and two approved ALS drugs in human neuroglioma cells transfected with wild-type, A4V and G93A SOD1. We found that human cells require higher dose of compounds to induce neuroprotection compared to mouse neuronal cells. MR6-26-2 was found to be the best compound to redeem cell viability of several fALS mutants tested here with negligible toxicity to the wild-type SOD1. Synergistic association of drugs targeting different mechanisms have been reported as a strategy to improve therapeutic effects on ALS^41^. Combined therapies of ebselen-based compounds/ALS drugs showed no synergistic improvement in in vitro neuroprotective activity compared to single-compound treatments. Due to the different degree of response to drug dosage and disease progression from ALS-associated mutants between human (A4V) and mouse (G93A), we validated mode of action of ebselen-based compounds in mouse model’s mutant by solving co-crystalised structures of G93A SOD1. These results confirm that these compounds stabilise dimer interface at Cys111 in both A4V and G93A SOD1 with akin poses. Targeting Cys111 is a promising strategy to stabilise human SOD1 proteins. Agar and colleagues have demonstrated that cross-linking Cys111 effectively stabilises SOD1 dimer structure with significant potential for exploitation for an effective therapy^42^. This is also reflected in MR6-26-2 ability to reduce the aggregates in all of the fALS SOD1 mutants (A4V, G93A, H46R and D90A) cells used here by stabilising the mutants SOD1 dimer. Given their effectiveness to a range of mutants tested here, our compounds are likely to be effective to most if not all the destabilising SOD1 mutants linked to ALS.

To demonstrate the on-target engagement of ebselen, MR6-8-2 and MR6-26-2, in human neuronal cells, we used a BiFC assay, based on the complementation of two nonfluorescent fragments of the fluorescent tag on SOD1, to monitor the dimerization of SOD1 in living human neuroglioma cells^28,29^. This assay proved to be a powerful tool to monitor aggregation propensity resulting from unstable dimer formation due to A4V mutation. The effects of ebselen and MR6-8-2 and MR6-26-2, to reduce formation of cellular A4V SOD1 aggregates have been visualised by fluorescence microscopy. Although based on cell viability assays, ebselen, MR6-8-2 or MR6-26-2 treatment at 50 μM seems more toxic to the human cells than 25 μM, neuroprotection through the reduction in aggregation propensity was more pronounced at 50 μM in the surviving cells compared to 25 μM. This is worth noting that the surviving cells under consideration are the transfected cell population which makes cell toxicity less of an issue, so the BiFC assay was performed at 50 μM of the compounds for aggregates proportion analysis. By using variety of different controls, we were able to demonstrate that the BiFC assay can reliably detect the protein aggregates and allows to capture the restoration of WT phenotype following drug-treatment. This is a significant improvement from previously published controls^29^. For example, absence of fluorescence in cells transfected with V_N_-SOD1 and empty V_C_ vectors or with empty V_N_ and SOD1-V_C_ ruled out the possibility of self-assembly of V_N_ and V_C_ fragments demonstrating that the uniform fluorescence when detected in the cells is resulting from true dimerization of SOD1 monomers and not due to the self-fusion of the two fragments. The use of background mutant C6S^27^, as positive control, displayed uniform fluorescence, consistent with dominant higher melting temperature species observed with Ebselen, further validating the BiFc assay for detection of stable dimers. The cells transfected with wild-type SOD1 showed aggregation-free uniform fluorescence throughout the cytosol indicating a proper dimerization of wild-type protein. In contrast, mutant SOD1 variants displayed high proportion of the cells with SOD1 inclusions arising due to misfolded SOD1. The cells with A4V SOD1 displayed highest percentage of ≤ 5 as well as > 5 inclusions per cell followed by G93A, H46R and D90A. However, majority of the cells expressing H46R (~ 40%) and D90A (60%) were found to be without inclusions (similar to wild type) compared to A4V (20%), consistent with much slower disease progression in H46R and D90A fALS patients. Interestingly, this percentage of cells with normal phenotype corresponds very well to the pathological effects of each mutation and the severity of symptoms with A4V being the most aggressive followed by the G93A and then the slower progressing D90A and H46R variants that are associated with a milder form of fALS and longer survival time despite H46R being a mutation of Cu binding residue^19^.

When treated with ebselen and two second-generation analogues, we observed that all three compounds were able to engage the SOD1 target by significantly reducing the propensity of aggregation as demonstrated by the reduction in the number of cells displaying inclusions as well as the number of inclusions within the cell. MR6-26-2 was found to exert the strongest effect not only in reducing the number of inclusions within the cells but also restoring the normal phenotype of the cells close to wild-type SOD1. Cell imaging profiles also showed that the compound-treated cells with mutant SOD1variants were healthy looking compared to the untreated cells, which appeared shrunk with deformed nuclei. This observation is consistent with our viability assay data that the treatment with ebselen-based compounds and particularly MR6-26-2 significantly increases the cell viability. Demonstration of engagement of these small molecules with the target protein in a cellular environment bodes well for early-stage drug discovery.

A single oral dose of MR6-26-2 at 30 mg/kg in female CD1 mice was shown to have 29% bioavailability with a short plasma half-life of 1.5 h with fast clearance. Whilst this represents a reasonable level of oral drug exposure, the overall DMPK characteristics represents a key area for future medicinal chemistry optimisation in pursuit of a drug candidate. Notwithstanding, the drug exposures were considered appropriate for a proof-of-concept study by in vivo experiments using SOD1^G93A^ mice. In our previous study, we have performed a screening of ebselen-derivative compounds for neuroprotection efficacy in the mouse neuroblastoma cells expressing G93A SOD1 mutant^25^. In addition, we have shown that ebselen also gave a partial neuroprotection in SOD1^G93A^ mice by delaying disease onset^25^. A key remaining question was whether selected ebselen-derivatives, MR6-8-2 and MR6-26-2, stabilise mutant SOD1 proteins and confer neuroprotection in the SOD1-fALS mouse model. Both ebselen-derivatives significantly reduced the misfolded SOD1 species and SOD1 oligomers with aberrant disulphide bonds in vivo in line with the stabilisation of mutant SOD proteins. Moreover, the neuroprotection property of orally administered MR6-26-2 was only slightly greater than ebselen by slowing disease onset of SOD1^G93A^ mice by approximately 15 days. Despite a limited effect on the survival time, MR6-26-2 conferred neuroprotection by maintaining neuromuscular junction from muscle denervation in SOD1^G93A^ mice clearly indicating the improvement in motor function. These data demonstrate better stabilising ability and in vitro cytoprotection of new compounds. To date, a number of small compound-based experimental therapies has been developed for SOD1-fALS models by targeting SOD1 structure, including copper loading^43^, targeting maturation^44^, and dimerisation^25^. Other strategies include targeting ER stress^45^ and inhibiting SOD1 oligomerisation and aggregation^46,47^. In comparison with previous studies, our newly developed compounds are orally available and well tolerated for a chronic administration in mice. Although detailed toxicology evaluation is required, our evidence offers these new compounds as a promising therapeutic option for SOD1-fALS. Moreover, considering that SOD1 mutations were found in up to 4% sALS cases^5^ and that SOD1 pathology is found in part of sporadic ALS patients^48–50^, our compounds likely to have therapeutic potential for at least some of the sporadic ALS cases too. It is clear that wild type and mutant SOD1 share some of the destabilized conformations^16,19,48^, which we propose can be stabilized by these and other similar compounds through interaction with Cys111.

## Materials and methods

### Synthesis of compounds

Ebselen, riluzole and edaravone were obtained from a commercial supplier (#E3520, #R116, and #443300 respectively Sigma Aldrich). Details of the synthesis and characterisation of MR6-8-2 and MR6-26*2 have been described in our previous report^25^.

### Bimolecular fluorescence complementation plasmids

The Venus-bimolecular fluorescence complementation (BiFC) plasmids containing wild-type SOD1 gene were generated by gene synthesis and cloning service of Genscript, USA. The sequences of a larger N-terminal fragment of Venus (V_N_) with residues 1–158 and a smaller C-terminal fragment (V_C_) with residues 159–239 were used for the construction of Venus-BiFC plasmids (Fig. 3A) as described previously^25^. Human SOD1 gene was cloned to the 3′end of 10xglycine linker connected with the V_N_ fragment (V_N_-SOD1) and the 5′end of the V_C_ fragment (SOD1-V_C_) as shown in Fig. 3A. V_N_-SOD1 and SOD1-V_C_ genes were cloned to pcDNA3.1(+) at HindIII/XhoI and AflII/XbaI restriction sites, respectively. The Venus-BiFC plasmids of A4V SOD1 were generated by site-direct mutagenesis service (Genscript, USA) using the Venus-BiFC plasmids of wild-type SOD1 as starting templates.

### Cell culture, transfection and cell viability assays

Human H4 neuroglioma cells (HTB-148, ATCC) and mouse Neuro2a neuroblastoma cells (CCL-131, ATCC) were cultured in Dulbecco’s modified Eagle’s medium (DMEM) (Gibco) supplemented with 10%v/v Foetal Bovine Serum (FBS) at 37 °C under humidified atmosphere with 5% CO_2_. Transfection of the Venus-BiFC SOD1 plasmids to H4 cells was carried out using Turbofect™ transfection agent (#R0533, Thermo Fisher Scientific) following protocols from the manufacture. Briefly, 1.5 × 10^5^ H4 cells suspended in 1 mL medium were seeded on a well of 4 compartment-glass bottom dish (Cellview™, Greiner Bio-one) and grown at 37 °C for 24 h. 0.25 µg Venus-BiFC plasmids of wild-type or A4V SOD1 homodimer comprised of equimolar concentrations of V_N_-SOD1 and SOD1-V_C_ were mixed with 1 µL Turbofect in 0.1 mL FBS-free DMEM and incubated at room temperature for 15 min before adding to seeded H4 cells. 25 and 50 µM ebselen, MR6-8-2 and MR6-26-2 diluted from 250 mM stock in DMSO were added to 24 h-transfected H4 cells and incubated for 24 h prior to live cell imaging. To determine cell viability, 2.5 × 10^4^ cells were seeded in 0.2 mL media in a 96-well plate (Corning Costar, #10695951, Thermo Fisher Scientific). Cell viability was measured either using MTS assay (CellTiter 96, #G3580, Promega) on a plate-reader spectrometer (SpectraMax Plus 384, Molecular Devices) or using CellTiter-GloR 2.0 Assay (#G9241, Promega) following the manufacturer’s protocol. This assay relies on the detection of luminescence signal as a function of ATP and hence metabolically active cells. BMG POLARstar Omega microplate reader was used for luminescence measurement. To avoid the interference of the antioxidant compounds (ebselen and its analogues) with the luciferase activity in the presence of oxygen, the drugs containing media was replaced with a fresh medium prior to the addition of the reagent.

Neuro2a cells were seeded on 6 well plate (#140685 Corning Costar) at 5.0 × 10^4^/well, and transfected with human wild-type or G93A SOD1 expressing plasmid^25^ using Lipofectamine 2000 (Thermo Fisher Scientific) according to the manufacturer’s instructions. After 6 h of transfection, the medium was replaced with a fresh differentiation medium [DMEM supplemented with 2%v/v FBS and 2.5 mM N6,2′-O-dibutyryladenosine-3′,5′-cyclic monophosphate (Nacalai Tesque, Kyoto, Japan)] containing 10 µM MR6-26-2, 10 µM riluzole ((#A2423, Tokyo Chemical Industry Co., Ltd., Tokyo, Japan), or 10 µM edaravone (#16509502, FUJIFILM Wako Pure Chemical Corp., Osaka, Japan). The cells were incubated at 37 °C under humidified atmosphere with 5% CO_2_ for 24 h. After the incubation, the cells were subjected to immunoblotting as previously described^51^.

### Laser scanning confocal fluorescence microscopy of live transfected cells

Human H4 neuroglioma cells cultured in glass-bottom dishes were transfected with Venus fragments as described above using Turbofect transfection reagent. Transfected H4 cells were stained with 5 µM DRAQ5 nuclear stain (ab108410, Abcam) and incubated at 37 °C for 5 min prior to cell imaging. Live cell imaging was performed on cells maintained in a humidified incubator (37 °C, 5% CO_2_) using a Zeiss LSM 710 microscope. To obtain large field of view for analysis, images were collected using a 4 × 4 tiles with a 20 × Fluar 0.8 NA (Carl Zeiss, Jena, Germany) at 1024 × 1024 pixels. Excitation of Venus fluorescent protein (VFP) was carried out with 514-nm argon laser while DRAQ5 was excited at 633 nm with a HeNe laser. Emitted light was collected through appropriate filters to eliminate any spill-overs. Image acquisition and processing were carried out using ZEN 2.1 software (Carl Zeiss, Jena Germany).

### Quantification of hSOD1 inclusions

Following the drug treatment (24 h) of transfected cells and nuclear staining with DRAQ5 for 5 min the tiled images were generated as described above. For each condition (WT, A4V, ebselen, MR6-8-2 and MR6-26-2) at least 200 transfected cells per imaging experiment were analysed, scored based on hSod1 inclusions pattern, and classified into 3 groups: cells without inclusions, ≤ 5 or > 5 inclusions^29^. Fiji software^52^ was used to determine minimum and maximum diameter of cells and inclusions within the cells in pixel units. These cell/inclusion sizes were then used to set parameters in Cell Profiler^53^. The pipeline was adapted from the Speckle Counter example available from CellProfiler (https://cellprofiler.org/) for the batch analysis of the cells with or without inclusions. The low fluorescent cells missed by the cell profiler were adjusted by manual counting.

### G93A SOD1 production and purification

We expressed G93A SOD1 in BL21(DE3) *E. coli* using the pET303C plasmid containing G93A human SOD1 gene as previously described^54^. The bacteria were cultured at 37 °C in LB broth and induced at 18 °C for 16 h by adding 0.5 mM IPTG and 0.3 mM ZnSO_4_ after optical density at 600 nm was between 0.6 and 0.8. G93A SOD1 was purified using protocol described previously^25^. Bacteria cell lysate was precipitated out using 2.5 M ammonium sulphate and loaded to Phenyl Sepharose column (GE Healthcare). G93A SOD1 was eluted in 1.00–1.75 M ammonium sulphate fractions and further purified by size-exclusion chromatography on a Superdex 200 16/600 column (GE Healthcare) eluted with 20 mM Tris pH 7.4, 150 mM NaCl. SOD1 concentration were quantified by 280 nm absorption using an extinction coefficient of 5,500 M^−1^ cm^−1^. The protein was snap-frozen in liquid nitrogen and stored at − 80 °C prior to doing experiments.

### SOD1 crystallography

SOD1 crystallization was carried out using the protocol that previously described in our publication^25^. Briefly, 10 mg/mL G93A SOD1 was incubated with fivefold molar excess of compounds and mixed with equal volume of reservoir solution. Reservoir solution was made of 100 mM sodium acetate pH 4.7, 150 mM NaCl and 2.4–2.7 M ammonium sulphate. Macro-seeding was done by adding a few pieces of SOD1 crystals into crystallization drops after 3-day incubation at 19 °C. Mature crystals grew within a week at 19 °C and cryo-protected in Paratone Oil (HR2-861, Hampton Research). Frozen crystals were diffracted at 100 K using 0.9800 Å X-rays on I24 at Diamond Light Source, UK. Diffraction images were indexed and integrated using DIALS^55^ and scaled using Aimless^56^ in CCP4i2 suite. Phases were solved by molecular replacement using a starting model of G93A SOD1 structure (PDB:2WKO) in MolRep^57^. Protein structures were refined in Refmac5^58^ and manually modelled in COOT^59^. Ligand models of ebselen and other compounds were produced in AceDRG^60^ and manually added into corresponding electron density in COOT.

### Survival and behavioral experiments of the mice

Transgenic mice expressing human SOD1^G93A^ with C57BL/6 background (B6.Cg‐Tg(SOD1*G93A)1Gur/J) (RRID: IMSR_JAX:004435) were prepared for in vivo experiments using our published protocol with determination of the times for disease onset and end-stage were previously^25,61^. The female SOD1^G93A^ mice were randomly divided into two groups that fed with a powdered CE-2 diet (CLEA Japan Inc., Tokyo, Japan) in the presence or absence of 0.016% MR6-8-2 or MR6-26-2 using a feeder for powder diet from 70 days of age to the end-stage under the same conditions as previously used for ebselen^25^. The diet was replenished three times per week, and the mice had free access to their assigned diet and deionized water. The body weight of the mice and clasping signs were measured every week. Disease onset was determined by the time when the mice reached maximum body weight. The clasping signs were scored as 0 (no sign), 1 (one hindlimb partially retracted), 2 (both hindlimb partially retracted), or 3 (hindlimbs were entirely retracted), as described elsewhere^62,63^. Rotarod tests were performed every two weeks, as previously described^61,62^. Briefly, the mice were placed on the rotating rods, which accelerated from 0 to 30 rpm for 5 min with 15 min interval between each trial. The longest latencies to fall from the rotating rods out of three trials were scored. All behavioral experiments were conducted under non-blinded conditions. The mice were housed in the specific pathogen-free (SPF) environment (with a 12 h light‐dark cycle at 23 ± 1 °C and 50 ± 5% humidity) and treated in compliance with the requirements of the Animal Care and Use Committee, Nagoya University. The experiments using genetically modified mice were approved by the Animal Care and Use Committee and the recombinant DNA experiment committee of Nagoya University. All methods used for transgenic mice experiments were in accordance with relevant guidelines and regulations and all methods are reported in accordance with ARRIVE guideline.

### Immunoblotting and immunofluorescence analyses of mouse spinal cords

Immunoblotting and immunofluorescence analyses were performed as described previously^62^. For the analysis of SOD1 oligomers with aberrant disulfide bonds (SOD1 S–S oligomers), sodium dodecyl sulfate–polyacrylamide gel electrophoresis was performed under non-reducing conditions^64^. For staining of misfolded SOD1, monoclonal antibody C4F6—raised against the ALS variant G93A of SOD1 and that has been shown to recognise a misfolded conformation shared by many ALS mutants of SOD^65^ (Medimabs, Montreal, Quebec, Canada) was used at a 1:250 dilution. Other primary antibodies used for the study were anti-β-actin (1:5,000, #A5441, RRID: AB_476744, Sigma-Aldrich), anti-p62 (1:1,000 for immunoblotting, 1:500 for immunohistochemistry, #PM045, RRID: AB_2169714, Medical and Biological Laboratories (MBL), Nagoya, Japan), anti-polyubiquitin (1:1,000 for immunoblotting, 1:500 for immunohistochemistry, #D058‐3, RRID: AB_592937, MBL), and anti-choline acetyltransferase (ChAT) ((1:100 for immunohistochemistry, #AB144P, RRID:AB_2079751, EMD Millipore Corp., Billerica, MA, USA). Rabbit anti-human SOD1 antibody was raised in our laboratory against a recombinant human SOD1 peptide (aa 24–37) and purified with protein A^66^.

### Neuromuscular junction (NMJ) analysis

After the mouse was deeply anesthetized and perfused with PBS, tibialis anterior (TA) muscle was dissected and briefly fixed in 4% paraformaldehyde at room temperature for 30 min. The fixed TA muscle was embedded in the Tissue Tek OCT compound (Sakura Finetek Japan Co., Ltd., Tokyo, Japan) and stored at – 80 °C until use. The TA muscle was sectioned at 30 µm thickness, and stained using α-bungarotoxin (BTX) Alexa Fluor 488 conjugate (1 µg/mL, #B13422, Thermo Fisher Scientific), anti-synaptophysin antibody (1:50, #ab16659, RRID: AB_44319, Abcam), and SMI-312 antibody (1:250, #837904, RRID: AB_2566782, Biolegend, San Diego, CA, USA) as previously described^67^. The muscle sections were analyzed by confocal microscopy, and co-localisation ratio of BTX and synaptophysin was quantified as innerved NMJ ratio for 30 NMJs each from three mice.

### Statistics

Data from the MTS and ATP assays were analysed using one-way ANOVA followed by the post-hoc Dunnet’s multiple comparison t-test in GraphPad Prism 8 software. In vitro BiFC based cell imaging data were also analyzed using GraphPad Prism 8 software and expressed as mean values SD or SEM of at least 3 independent experiments. Statistical differences of mutant SOD1 versus drug treated conditions were performed using one-way ANOVA with Dunnet’s multiple comparisons. Fractions of inclusions (0 inclusions; ≤ 5 and > 5) prevalent in each non-treated hSOD1 mutants (A4V, G93A, H46R and D90A) were compared using 2-way ANOVA with Tukey’s multiple comparison test. Onset or survival curves of mice were analyzed by a Log-rank test. All the semi-quantitative immunoblotting data and the number of inclusion bodies observed in anterior horns of at least three mice were analyzed by Welch’s *t*-tests. Significance levels were presented as ***p < 0.001 and **** *p* < 0.0001, where the precise *p*-values were not indicated.

### Ethics declarations

The experiments using genetically modified animals and organisms were approved by the Animal Care and Use Committee and recombinant DNA experiment committee of Nagoya University.

### Supplementary Information

Supplementary Information.

## Data Availability

All data needed to evaluate the conclusions in the paper are present in the paper and/or the Supplementary Materials.

## Code availability

Accession code(s): The atomic coordinates have been deposited in the Protein Data Bank (PDB ID codes: 7T8E, 7T8F, 7T8G and 7T8H).
