# Supplementary material for: Ebselen analogues delay disease onset and its course in fALS by on-target SOD-1 engagement
Source: Sci Rep. 2024 May 27;14:12118. doi: 10.1038/s41598-024-62903-5 (PMC11130262; doi:10.1038/s41598-024-62903-5)
Supplement: Supplementary file 1 — Supplementary Information. [file 41598_2024_62903_MOESM1_ESM.docx]

**Supplementary Information**

**Ebselen Analogues delay disease onset and its course in fALS by on-target SOD-1 engagement**

Seiji Watanabe^a,1^, Kangsa Amporndanai^b,1,†^, Raheela Awais^c,1^, Caroline Latham^c^_,_ Muhammad Awais^d^, Paul M. O’Neill^e*^, Koji Yamanaka^a,f,g*^, S. Samar Hasnain^b,*^

^a^ Department of Neuroscience & Pathobiology, Research Institute of Environmental Medicine, Nagoya University, Furo-cho, Chikusa-ku, Nagoya, 464-8601, Japan.

^b^ Molecular Biophysics Group, Department of Biochemistry and System Biology, Institute of System, Molecular and Integrative Biology, Faculty of Health and Life Sciences, University of Liverpool, Liverpool, L69 7ZB, United Kingdom.

^c^ School of Life Sciences, Faculty of Health and Life Sciences, University of Liverpool, Liverpool, L69 7ZB, United Kingdom.

^d^ Department of Molecular and Clinical Cancer Medicine, Institute of System, Molecular and Integrative Biology, University of Liverpool, Liverpool, L69 3GE, United Kingdom.

^e^ Department of Chemistry, Faculty of Science and Engineering, University of Liverpool, Liverpool, L69 7ZD, United Kingdom.

^f^ Institute for Glyco-core Research (iGCORE), Nagoya University, Nagoya, Japan.

^g^ Center for One Medicine Innovative Translational Research (COMIT), Nagoya University, Japan.


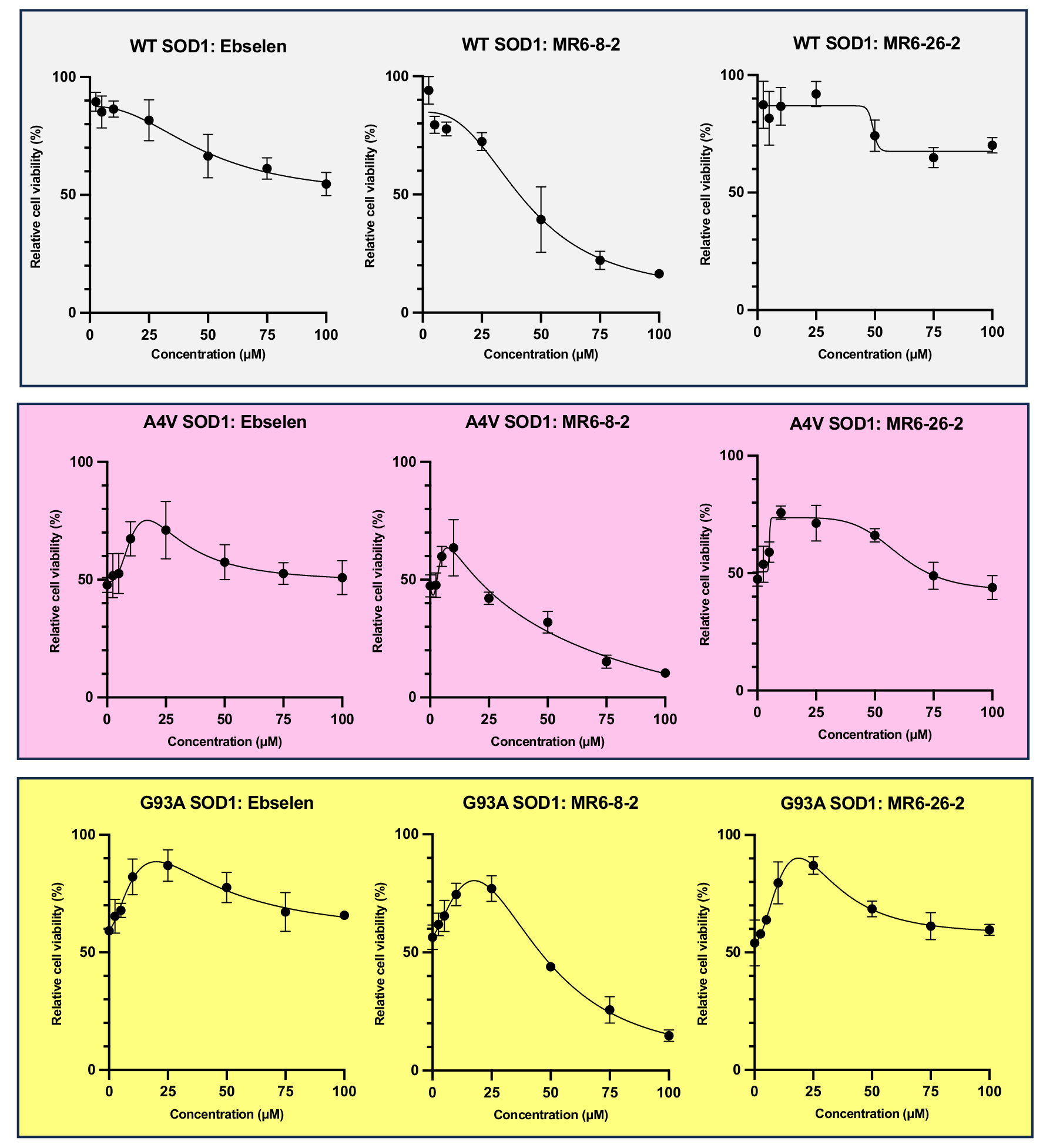


**Supplementary Figure S1.** Dose-response curves of Ebselen, MR6-8-2, and MR6-26-2 in human H4 cells expressing wild-type, A4V and G93A SOD1.


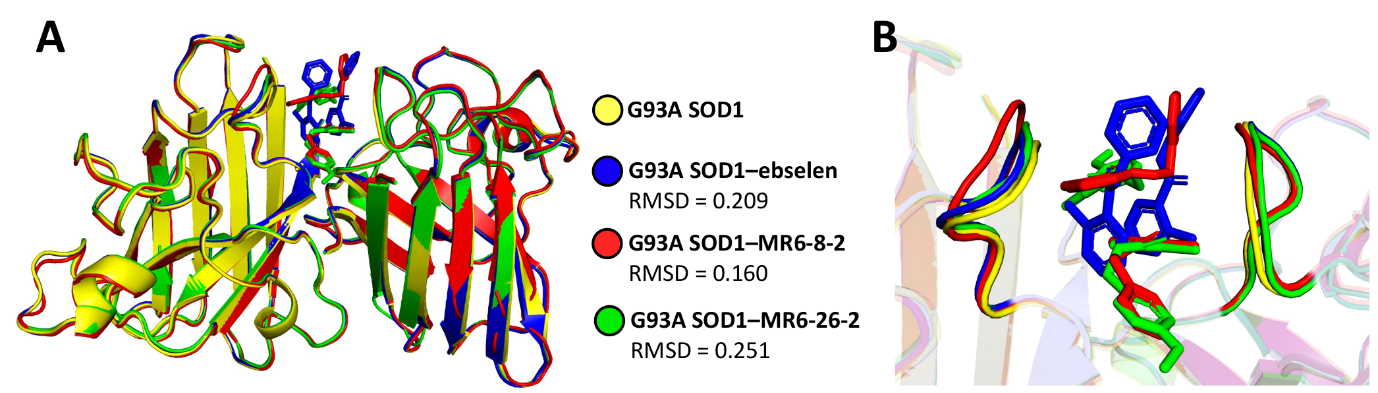


**Supplementary Figure S2.** Comparison of crystal structures of G93A SOD1 with/without compounds bound. **A** Superimposed structures and RMSD values compared to ligand-free G93A SOD1. **B** Close-up view of binding site of ebselen and derivatives at dimer interface. Protein structures and ligands of ligand-free, ebselen, MR6-8-2, and MR6-26-2 bound G93A SOD1 are coloured in yellow, blue, red and green, respectively.


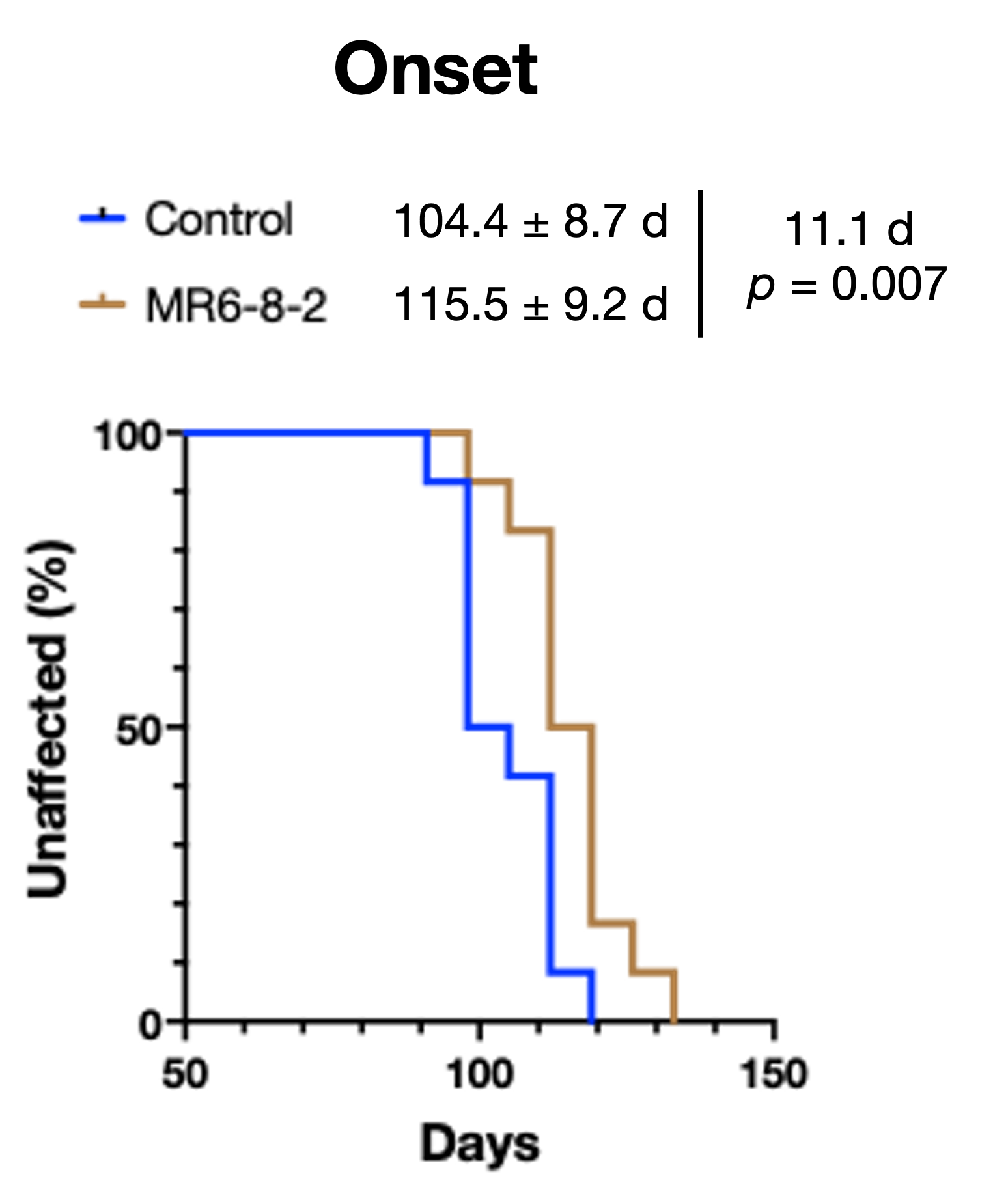


**Supplementary Figure S3.** MR6-8-2 delayed the disease onset of SOD1^G93A^ mice. Onset curves of the control or MR6-8-2 treated female SOD1^G93A^ mice plotted over time (n = 12 each). The mean ages for onset are shown with SD.

**Supplementary Figure S4.** Time course of body weight **A** and rotarod score **B** of the control or MR6-26-2 treated female SOD1^G93A^ mice (n = 20 each). The data were expressed as means with SD.

**Supplementary Figure S5.** Full membrane images for cropped immunoblotting images in Figure 5D (A) and F (B). All the membranes were cut before incubation with the indicated antibodies.

**Supplementary Figure S6.** Full membrane images for cropped immunoblotting images in Figure 6C (A) and H (B). The cropped area for soluble p62 was indicated by the red box. Full membrane images of the replicates used for quantification in Figure 6I–L were also shown in (C). All the membranes were cut before incubation with the indicated antibodies.

**Table S1. ﻿Crystallographic data collection and refinement statistics of G93A SOD1 with ebselen and derivative compounds crystals in P2_1_ space group**

| **Parameter/ligand** | **G93A SOD1** | **G93A SOD1- Ebselen** | **G93A SOD1- MR6-8-2** | **G93A SOD1- MR6-26-2** |
| --- | --- | --- | --- | --- |
| **Data collection** |  |  |  |  |
| Space group | P2_1_ | P2_1_ | P2_1_ | P2_1_ |
| Cell dimensions |  |  |  |  |
| a, b, c (Å) | 38.74, 68.21, 51.08 | 38.67, 67.99, 50.42 | 38.84, 67.79, 51.16 | 38.54, 68.48, 49.96 |
| α, β, γ (°) | 90.00, 105.63, 90.00 | 90.00, 105.61, 90.00 | 90.00, 106.29, 90.00 | 90.00, 105.02, 90.00 |
| Resolution (Å)* | 49.19-1.40  (1.42-1.40) | 67.99-1.40  (1.42-1.40) | 37.28-1.35  (1.37-1.35) | 68.48-1.50  (1.53-1.50) |
| R_merge_* | 6.5 (54.2) | 10.9 (49.6) | 4.3 (50.2) | 5.2 (50.5) |
| I/σI* | 8.7 (1.8) | 5.4 (1.6) | 11.1 (1.8) | 11.2 (2.4) |
| CC1/2 (%)* | 0.994 (0.563) | 0.976 (0.576) | 0.996 (0.791) | 0.998 (0.574) |
| Completeness (%)* | 99.6 (99.2) | 99.4 (93.5) | 97.3 (88.8) | 99.3 (98.0) |
| Redundancy* | 3.2 (2.8) | 3.0 (2.0) | 3.0 (2.0) | 3.1 (2.7) |
| **Refinement** |  |  |  |  |
| No. reflections | 50,157 | 49,165 | 54,294 | 39,919 |
| R_work_/R_free_ | 19.9/22.9 | 20.3/22.5 | 18.9/20.2 | 18.6/22.4 |
| No. atoms |  |  |  |  |
| Protein | 2,206 | 2,197 | 2,212 | 2,216 |
| Ligand/ion  Water | 0/24  240 | 32/14 229 | 34/28 328 | 38/24 223 |
| B-factors |  |  |  |  |
| Protein | 19.66 | 14.89 | 19.77 | 18.02 |
| Ligand/ion | -/31.14 | 27.27/27.31 | 25.34/27.18 | 36.28/18.63 |
| Water | 29.59 | 25.20 | 32.84 | 27.91 |
| R.M.S. dev. |  |  |  |  |
| Bond lengths (Å) | 0.0184 | 0.0176 | 0.0118 | 0.0140 |
| Bond angles (°) | 1.713 | 1.690 | 1.502 | 1.550 |
| PDB code | 7T8E | 7T8F | 7T8G | 7T8H |

*Values in parentheses are for the highest-resolution shell.
